# Supplementary material for: N-Acetyl-l-phenylalanine Racemization during TBTU Amidation: An In-Depth Study for the Synthesis of Anti-Inflammatory 2-(N-Acetyl)-l-phenylalanylamido-2-deoxy-d-glucose (NAPA)
Source: Molecules. 2023 Jan 6;28(2):581. doi: 10.3390/molecules28020581 (PMC9863357; doi:10.3390/molecules28020581)
Supplement: Supplementary file 1 [file molecules-28-00581-s001.zip › molecules-2120896-supplementary.pdf]

Supporting information:

# *N*-Acetyl-L-phenylalanine Racemization during TBTU Amidation: An In-Depth Study for the Synthesis of Anti-Inflammatory 2-(*N*-Acetyl)-L-phenylalanylamido-2-deoxy-D-glucose (NAPA)

**Elisa Sturabotti [1,*], Fabrizio Vetica [1], Giorgia Toscano [1], Andrea Calcaterra [2,*], Andrea Martinelli [1], Luisa Maria Migneco [1] and Francesca Leonelli [1,*]**

[1] Department of Chemistry, Sapienza University of Rome, P.le Aldo Moro 5, 00185 Rome, Italy; fabrizio.vetica@uniroma1.it (F.V.); toscano.1676782@studenti.uniroma1.it (G.T.); andrea.martinelli@uniroma1.it (A.M.); luisamaria.migneco@uniroma1.it (L.M.M.)
[2] Department of Chemistry and Technology of Drugs, Sapienza University of Rome, P.le Aldo Moro 5,
00185 Rome, Italy
* Correspondence: elisa.sturabotti@uniroma1.it (E.S.); andrea.calcaterra@uniroma1.it (A.C.); francesca.leonelli@uniroma1.it (F.L.)

## NMR spectra (400 MHz $CDCl_3$)

All $^{1}H$ and $^{13}C$ NMR spectra were recorded on a Bruker AVANCE-400, operating at 400.13 MHz for the proton and 100.61 MHz for the carbon spectra. $CDCl_3$ signal is at 7.26 ppm.

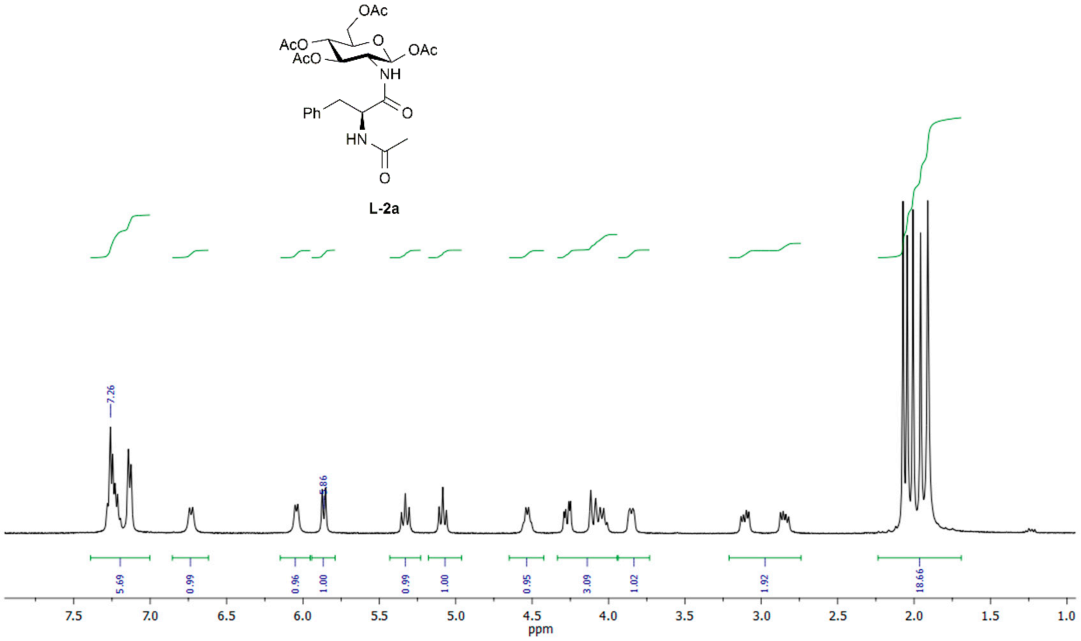


**Figure S1.** $^{1}H$ NMR for compound **L-2a**. L-H-1 at 5.86 ppm.

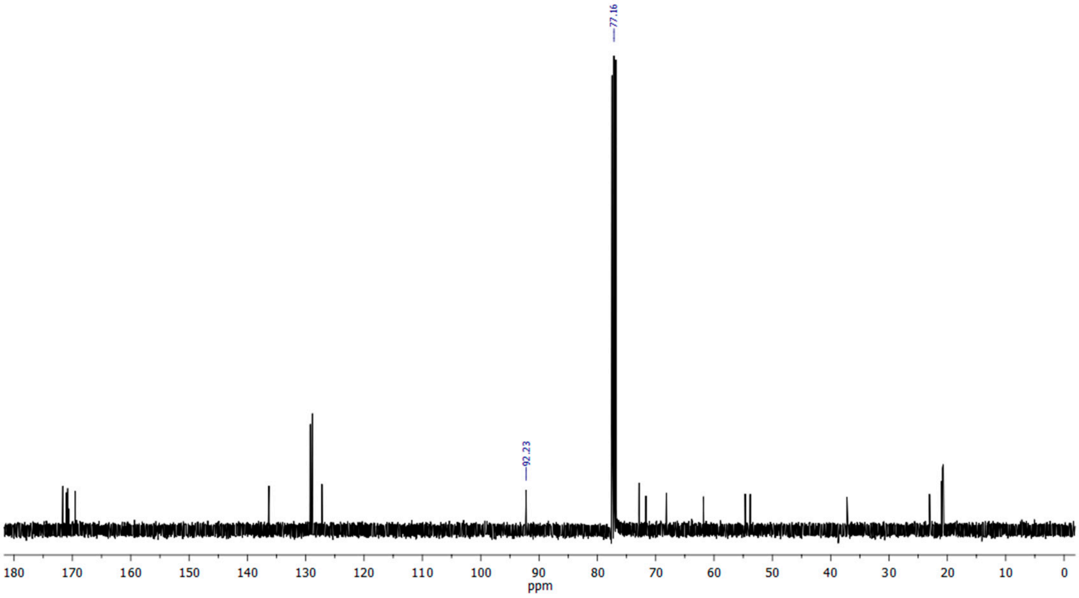


**Figure S2.** $^{13}$C NMR for compound **L-2a**.

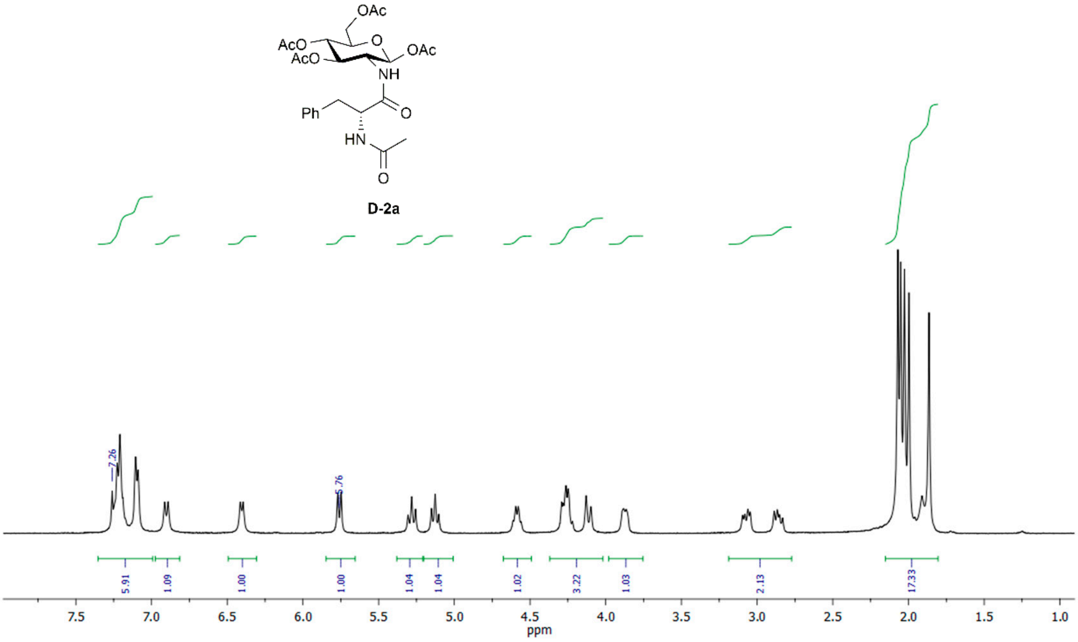


**Figure S3.** $^{1}$H NMR for compound **D-2a**. D-H-1 at 5.76 ppm.

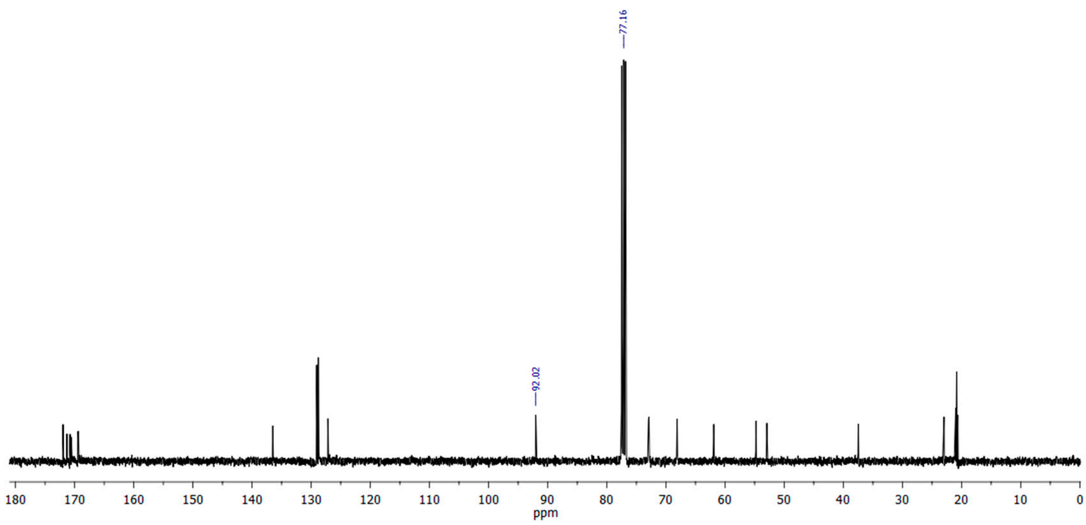


**Figure S4.** $^{13}C$ NMR for compound **D-2a**.

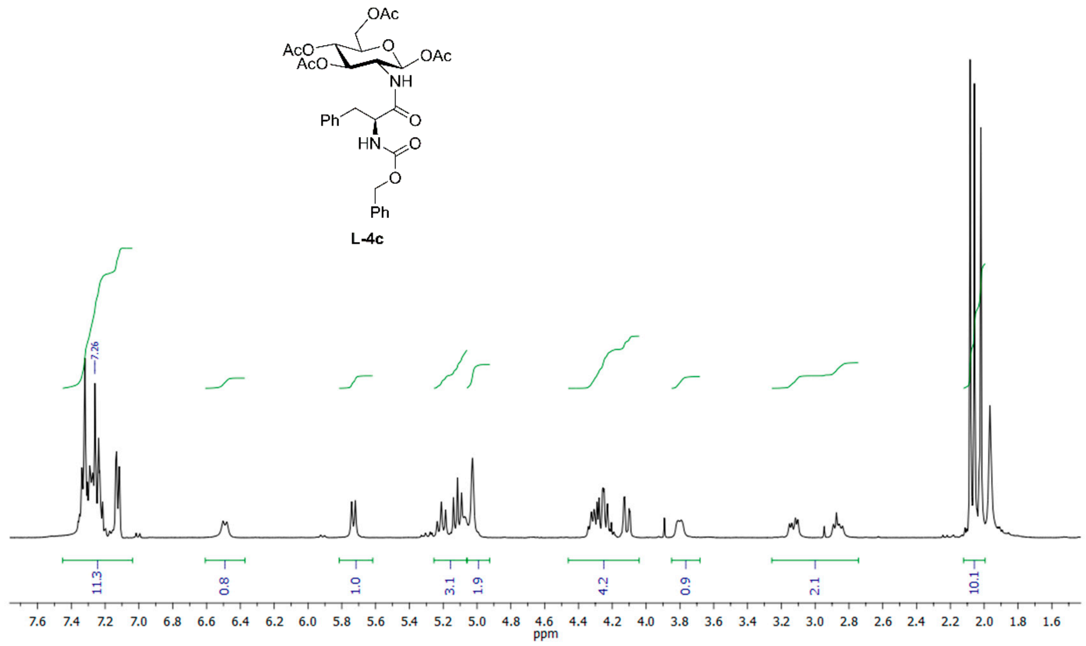


**Figure S5.** $^{1}H$ NMR for compound **L-4c**.

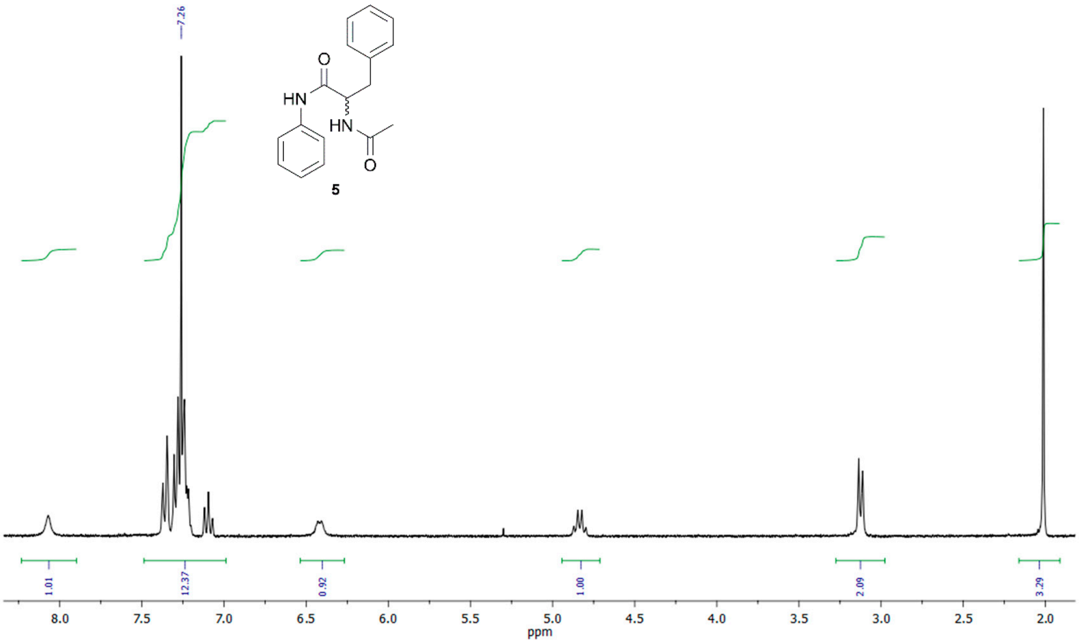


**Figure S6.** $^{1}$H NMR for compound **5**.

## HPLC chromatogram

Column: Chiralpak, IA, Daicel chemical industries (H-iPrOH-95-5, 1 ml/min).

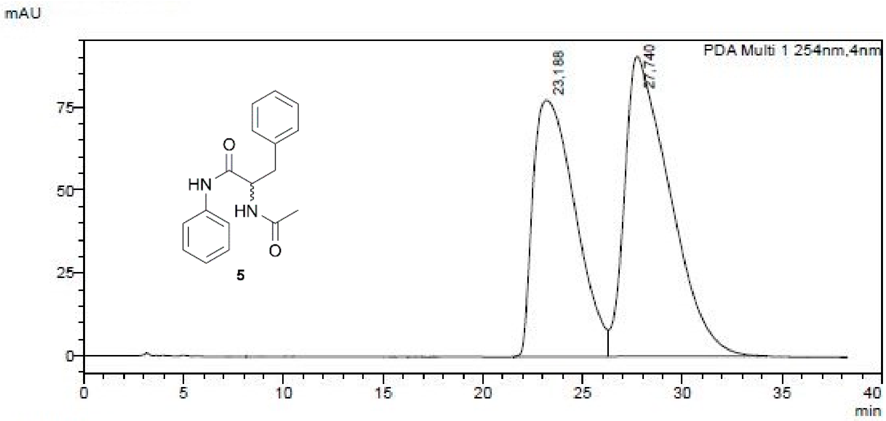


<Peak Table>

PDA Ch1 254nm

| Peak# | Ret. Time | Area | Height | Area% |
|---|---|---|---|---|
| 1 | 23,188 | 11376371 | 77277 | 44,280 |
| 2 | 27,740 | 14315807 | 90342 | 55,720 |
| Total | | 25692178 | 167619 | 100,000 |

**Figure S7.** Chromatogram of compound **5** from pure *N*-Ac-L-phe with TBTU/DIPEA.

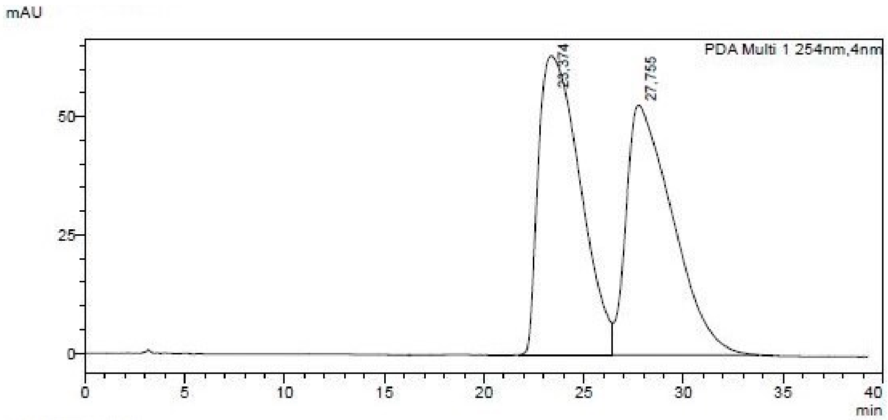


**<Peak Table>**

PDA Ch1 254nm

| Peak# | Ret. Time | Area | Height | Area% |
|---|---|---|---|---|
| 1 | 23,374 | 9185655 | 63175 | 51,737 |
| 2 | 27,755 | 8569018 | 52814 | 48,263 |
| Total | | 17754673 | 115989 | 100,000 |

**Figure S8.** Chromatogram of compound **5** from pure N-Ac-D-phe with TBTU/DIPEA.

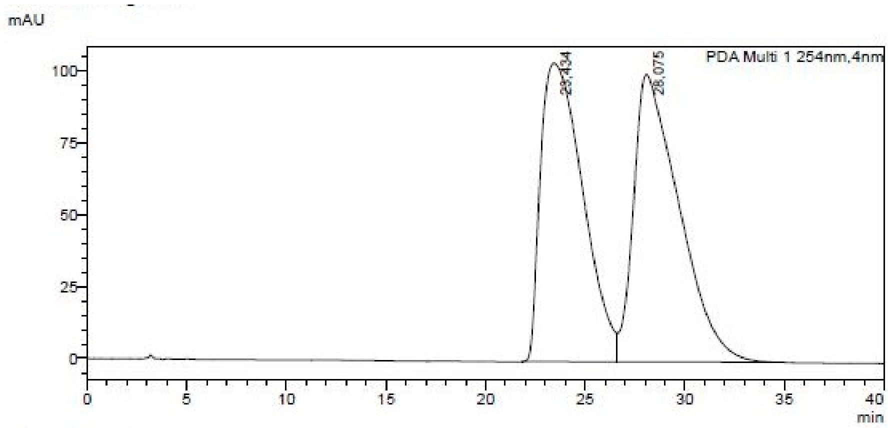


**<Peak Table>**

PDA Ch1 254nm

| Peak# | Ret. Time | Area | Height | Area% |
|---|---|---|---|---|
| 1 | 23,434 | 15355208 | 103812 | 49,085 |
| 2 | 28,075 | 15927992 | 100077 | 50,915 |
| Total | | 31283201 | 203889 | 100,000 |

**Figure S9.** Chromatogram of compound **5** from N-Ac-L-phe:N-Ac-D-phe=1:1 with TBTU/DIPEA.

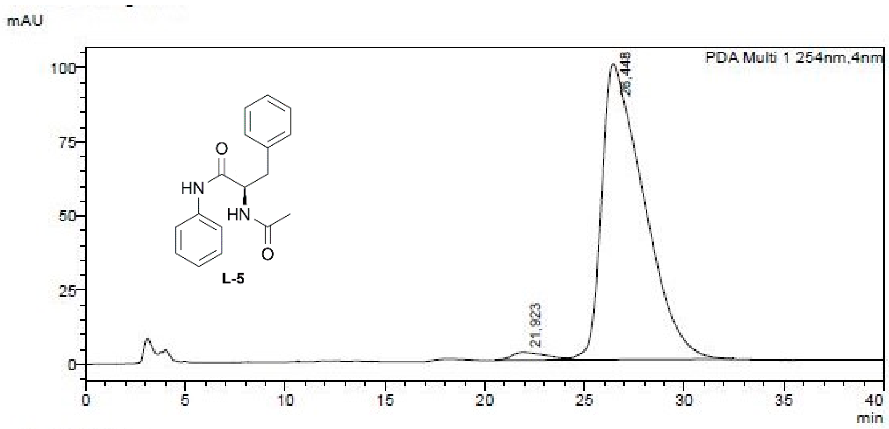


**<Peak Table>**

PDA Ch1 254nm

| Peak# | Ret. Time | Area | Height | Area% |
|---|---|---|---|---|
| 1 | 21,923 | 314557 | 2620 | 2,096 |
| 2 | 26,448 | 14690309 | 99582 | 97,904 |
| Total | | 15004866 | 102203 | 100,000 |

**Figure S10.** Chromatogram of compound **L-5** from pure N-Ac-L-phe and pyridine.
